# Expression of Kielin/chordin-like protein is regulated by BMP-2 in osteoblasts

**DOI:** 10.1016/j.bonr.2024.101793

**Published:** 2024-07-20

**Authors:** Kazuki Toba, Atsushi Yamada, Kiyohito Sasa, Tatsuo Shirota, Ryutaro Kamijo

**Affiliations:** a Department of Biochemistry, Graduate School of Dentistry, Showa University, Tokyo, Japan; b Department of Oral and Maxillofacial Surgery, Graduate School of Dentistry, Showa University, Tokyo, Japan

**Keywords:** BMP, Kcp, Osteoblasts

## Abstract

Bone morphogenetic protein (BMP), an osteoinductive factor, is a cytokine that induces osteoblast differentiation and mineralization, and expected to be applicable for hard tissue reconstruction. Kielin/chordin-like protein (Kcp), a member of the family of cysteine-rich proteins, enhances BMP signaling. The present study found that expression of *Kcp* in osteoblasts was induced by BMP-2 in a concentration- and time-dependent manner. Up-regulation of *Kcp* by BMP-2 was inhibited by Dorsomorphin, a SMAD signaling inhibitor. The involvement of up-regulation of *Kcp* by BMP-2 in induction of osteoblast differentiation by BMP-2 was also examined, which showed that suppression of *Kcp* expression by si *Kcp* partially inhibited induction of osteoblast differentiation and mineralization by BMP-2. Together, these results suggest that *Kcp* induced by BMP-2 functions to provide positive feedback for promotion of osteoblastic differentiation and mineralization by BMP-2 in osteoblasts.

## Highlights

• *Kcp* expression was induced in osteoblasts by BMP-2 in a concentration- and time-dependent manner.
• BMP-2-induced *Kcp* expression was regulated by SMAD signaling.
• Induction of *Kcp* partially regulated BMP-2-induced osteoblast differentiation.

## 1 Introduction

Bone morphogenetic proteins (BMPs) have important roles in embryogenesis, organogenesis, cell proliferation, and cell differentiation (Miyazawa et al., 2002). These proteins belong to the transforming growth factor-β (TGF-β) superfamily, with approximately 20 different BMP families identified and characterized thus far, and shown to regulate bone balance and osteogenic function by regulating osteoblast differentiation (Wan and Cao, 2005). BMPs are expected to be applied for hard tissue reconstruction, thus it is crucial to elucidate factors related to promotion of their activity for effective clinical applications (Zhu et al., 2022).

BMPs bind to the serine/threonine kinase receptors BMPR-I and II, four and three transmembrane receptors, respectively, for signal transduction. When BMPs bind to these receptors, BMPR-II phosphorylates BMPR-I (Ebendal et al., 1998), while activated BMPR-I phosphorylates the receptor-regulated Smads (R-Smads) Smad1, 5, and 8 (Gomez-Puerto et al., 2019). A phosphorylated R-Smad forms a heterotrimer with Smad4 and migrates into the nucleus to regulate expression of target genes. This SMAD-mediated BMP signaling is known to be inhibited by Dorsomorphin, which blocks the action of BMPR-I (Yu et al., 2008). It has also reported that BMP-2 activates members of the non-SMAD-mediated signaling pathway, including mitogen-activated protein kinase (MAPK), leading to induction of osteoblast differentiation (Chen et al., 2012).

Kielin/chordin-like protein (Kcp), which has 18 cysteine-rich domains and a high amino acid sequence homology to the BMP antagonist chordin, enhances BMP signaling by activating BMP receptors (BMPR-I) in the paracrine and promotes phosphorylation of intracellular Smads, while its physiological function is involved in attenuating renal fibrotic disease (Lin et al., 2005a). Recently, Kcp was shown to enhance BMP-2-induced osteoblast differentiation through SMAD signaling (Zhu et al., 2022; Nagasaki et al., 2023; Zhao et al., 2006). The present study was conducted to elucidate the regulatory mechanism of *Kcp* expression by BMP-2 in osteoblasts.

## 2 Materials and methods

### 2.1 Reagents

BMP-2 was purchased from R&D Systems (355-BM), BMP-4 from FUJIFILM Wako Pure Chemical Corporation (023–18,461), BMP-5 from R&D Systems (6176-BM), and BMP-7 from R&D Systems (5666-BP), Dorsomorphin was purchased from FUJIFILM Wako Pure Chemical Corporation (044–33,751). U0126 (MEK 1/2 inhibitor; 9903) was purchased from Cell Signaling, and SP600125 (JNK inhibitor; S1460) and SB203580 (p38 MAPK inhibitor; S1076) were purchased from Selleck.

### 2.2 Cell culture

MC3T3-E1, a mouse cranial crown-derived osteoblast cell line, was maintained in culture medium produced using 500 ml of α-MEM along with l-glutamine and phenol red medium (FUJIFILM Wako Pure Chemical Industries; 135–15,175), supplemented with 55 ml of fetal bovine serum (10 % FBS) (SIGMA-ALDRICH Corporation; 173,012) and 5 ml of penicillin-streptomycin (1 % penicillin-streptomycin) (Life Technologies Corporation, 15,240–062), at 37 °C in a CO_2_ incubator (5 % CO_2_, 95 % air). For collecting primary cultured osteoblasts, the skull of a one-day-old mouse was excised, then attached soft tissue was removed and placed in a 0.1 % collagenase/0.2 % dispase solution produced using 40 ml of culture medium supplemented with 40 mg of collagenase (0.1 % collagenase) and 80 mg of dispase (0.2 % dispase), then shaken at 37 °C for 5 min. After discarding the supernatant, fresh 0.1 % collagenase/0.2 % dispase solution was added, then shaking was continued at 37 °C for 10 min. The supernatant was obtained, and centrifuged and processed through a 40-μm cell strainer. Primary cultured osteoblasts were then maintained in culture medium at 37 °C in a CO_2_ incubator.

### 2.3 Kcp knockout mice

Kcp knockout mice (B6.Cg-KCP < tm1Ysas>/Rbrc, Accession No. CDB401K; https://large.riken.jp/distribution/mutant-list.html) were purchased from RIKEN BioResource Center (Ikeya et al., 2006). All protocols used for the animal experiments in this study were approved by the Showa University Animal Experiment Committee.

### 2.4 Quantitative RT-PCR

Total RNA was extracted from cells using TRIzol® Reagent (Life Technologies; 15,596,018), then cDNA was synthesized using ReverTra Ace® qPCR RT Master Mix (TOYOBO CO., LTD.; FSQ-201). Quantitative real-time PCR (95 °C for 1 min, followed by 40–50 cycles at 95 °C for 15 s and 60 °C for 30 s) was performed using THUNDERBIRD® Probe qPCR Mix (TOYOBO CO., LTD.; QPS-101). TaqMan™ IDs (Applied Biosystems) for gene expression assays were as follows: mouse *Gapdh* (Applied Biosystems; Mm99999915_g1), mouse *Kcp* (Applied Biosystems; Mm01159615_m1, Mm01163386_m1), mouse *Tnap* (Applied Biosystems; Mm00475834_m1), mouse *Runx2* (Applied Biosystems; Mm00501584_m1), and mouse *Sp7* (Applied Biosystems; Mm04209856_m1) (Aizawa et al., 2019).

### 2.5 Western blotting

MC3T3-E1 cells (5 × 10^5^ cells) were treated with a cell lysate sample buffer solution (1 ml) along with 3-Mercapto-1,2-propanediol (x4) (FUJIFILM Wako Pure Chemical Corporation; 196–16,142). Samples (25 μl) and marker proteins (25 μl) (Precision Plus Protein Western C Standards, BIORAD; 1,610,376) were separated into polyacrylamide gels using SDS-PAGE. After transfer, blocking was performed with 4 % skin milk (Difco Skim Milk, BD; 232,100) (Kinoshita et al., 2021). Smad5 (Smad5 D4G2 rabbit mAb, Cell Signaling TECHNOLOGY; 12,534), p-Smad1/5 (phospho-Smad1/5 Ser463/465; 41D10, rabbit mAb, Cell Signaling TECHNOLOGY; 9516), actin (SIGMA-ALDRICH; A5060), and anti-rabbit IgG horseradish peroxidase-linked whole donkey (GE Healthcare; NA934V) antibodies were used. Thereafter, reaction solution (ECL Prime Western Blotting Detection Reagent, Global Life Sciences Solutions Operations; RPN2232) treatment was performed and images were obtained using a VersaDoc IMAGING SYSTEM 5000 (BIORAD).

### 2.6 Knockdown by si *Kcp*

MC3T3E1 cells were transfected with RNAi max (Life Technologies Corporation; 13,778–150) for si *Kcp* in accordance with the protocol of the manufacturer. The respective oligos for *Kcp* were as follows: 5’-CCACAAUGGGCAGUCUUAUGGUCAU-3′ and 5’-AUGACCAUAAGACUGCCCAUUGUGG-3′.

### 2.7 ALP staining

MC3T3-E1 cells were introduced to si *Kcp* and seeded into 96-well plates (2 × 10^4^ cells/well), then cultured for 72 h in the presence or absence of BMP-2 (300 ng/ml). Cells were then fixed with 4 % paraformaldehyde (FUJIFILM Wako Pure Chemical Corporation; 163–20,145) for 30 min and washed with 1 X PBS. Next, they were stained with 10 ml of Tris-HCl buffer (200 mM, pH 8.5) containing 1 mg of naphthol AS-MX phosphate (Sigma-Aldrich) and 2.5 mg of Fast blue BB (Sigma-Aldrich). After washing with distilled water, observations were performed with a microscope.

### 2.8 ALP activity

MC3T3-E1 cells were cultured in 96-well plates and washed with 1 X PBS, then 1 % Nonidet P40 was added. After treatment at room temperature for one hour, the cells were disrupted with ultrasonic waves (Handy Sonic UR-21P). Next, 5 ml of ALP activity buffer produced using ALP solution buffer (1 mM MgCl_2_, 200 mM Tris-HCl) containing 24 mg of disodium *p*-nitrophenyl phosphate hexahydrate) was added to the lysate and absorbance (405 nm) was determined (Sasa et al., 2018).

### 2.9 Alizarin red staining

MC3T3-E1 cells were introduced to si *Kcp* and seeded into 96-well plates (2 × 10^4^ cells/well), then cultured for seven days in calcification culture medium, produced using 50 ml of culture medium with β-glycerophosphate (10 mM) and dexamethasone (10 nM), with the medium replaced every four days. The supernatant was collected and discarded, then cells were washed with 1 X PBS, fixed with 95 % methanol for 20 min at room temperature, and stained with an Alizarin red staining solution made with 1 g of Alizarin Red S (FUJIFILM Wako Pure Chemical Corporation; 011–01192) in 100 ml of distilled water (pH 6.3–6.5) for five minutes. After washing with distilled water, microscopic observations were performed. For quantitative analysis, the cell mixture was dissolved in a cetylpyridinium chloride solution, produced using 10 g of cetylpyridinium chloride (NAKARAI Chemical Corporation; 07905–05) in 100 ml distilled water, and absorbance at 570 nm was determined.

### 2.10 Histomorphometric analyses

Femora were obtained from Kcp knockout mice, then fixed in 70 % ethanol and subjected to Villanueva bone staining without decalcification (Ito Bone Histomorphometry Institute). Parameters related to bone structure were examined, including total bone volume per tissue volume (BV/TV, %), trabecular thickness (Tb.Th, μm), osteoid thickness (O·Th, μm), number of osteoblasts per bone surface (N.Ob/BS, N/mm), and number of osteoclasts per bone surface (N.Ob/BS, N/mm).

### 2.11 Statistical analysis

Data are expressed as the mean ± SD. Student's *t*-test was used for statistical analyses, with *p* < 0.05 considered to indicate significance. Experiments were performed at least twice.

## 3 Results

### 3.1 BMP-2 induces Kcp expression in concentration- and time-dependent manner

**Fig. 1:**
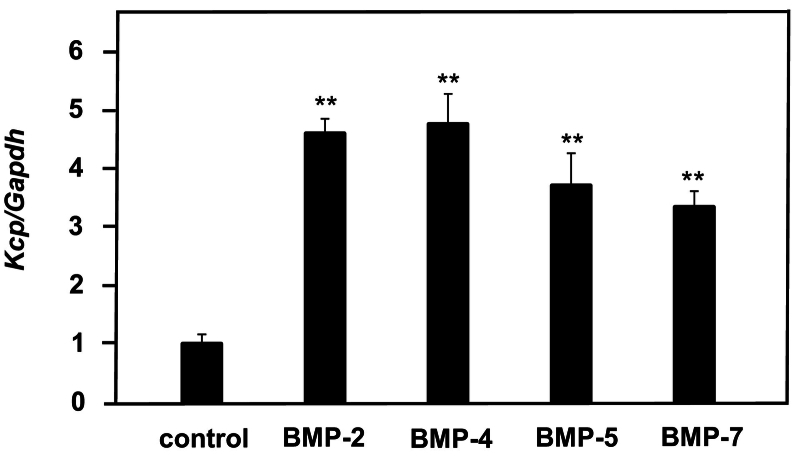
MC3T3-E1 cells were treated with 300 ng/ml of BMP-2, −4, −5 and − 7 for 24 h, then the expression level of *Kcp* was examined. Using a control *Kcp/Gapdh* value of 1, the relative ratio of *Kcp/Gapdh* after BMP treatment was determined. **p < 0.01 (Student's *t*-test).

**Fig. 2:**
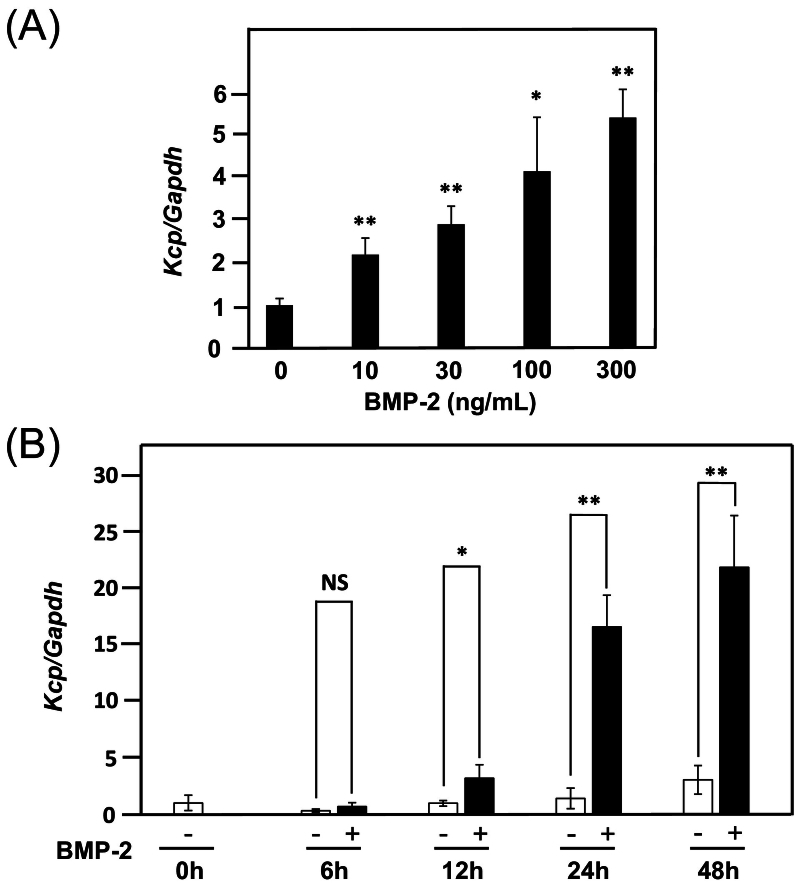
(A) Various concentrations (10, 30, 100, 300 ng/ml) of BMP-2 were applied to MC3T3-E1 cells for 24 h, then the expression level of *Kcp* was examined. Using a control *Kcp/Gapdh* value of 1, the relative ratio of *Kcp/Gapdh* when treated with BMP-2 at each concentration was determined. (B) Time course analysis of the effects of BMP-2 (300 ng/ml) on *Kcp* gene expression from 6 to 48 h. *p < 0.05, **p < 0.01 (Student's *t*-test). NS: not significant.

Kcp functions as an agonist to enhance BMP signaling (Lin et al., 2005a). Therefore, to examine the expression of *Kcp* when BMPs are applied, MC3T3-E1 cells were treated with 300 ng/ml of BMP-2, −4, −5, and − 7 for 24 h, then increases in expression were observed (Fig. 1). Similar experiments were conducted using mouse skull-derived primary cultured osteoblasts, with increased *Kcp* expression observed (Suppl. Fig. 1). These results indicated that BMPs induce *Kcp* expression in osteoblasts. Furthermore, when MC3T3-E1 cells were exposed to various concentrations of BMP-2 for 24 h, a concentration-dependent increase in *Kcp* expression was noted. As for the effects of BMP-2 treatment on *Kcp* for times ranging from 6 to 48 h, no increase in expression was observed after 6 h of treatment, while induction of expression was observed after 12 h (Fig. 2). It is thus suggested that BMP-2 induces *Kcp* expression in a concentration- and time-dependent manner.

### 3.2 BMP-2 induces Kcp expression through SMAD signaling

**Fig. 3:**
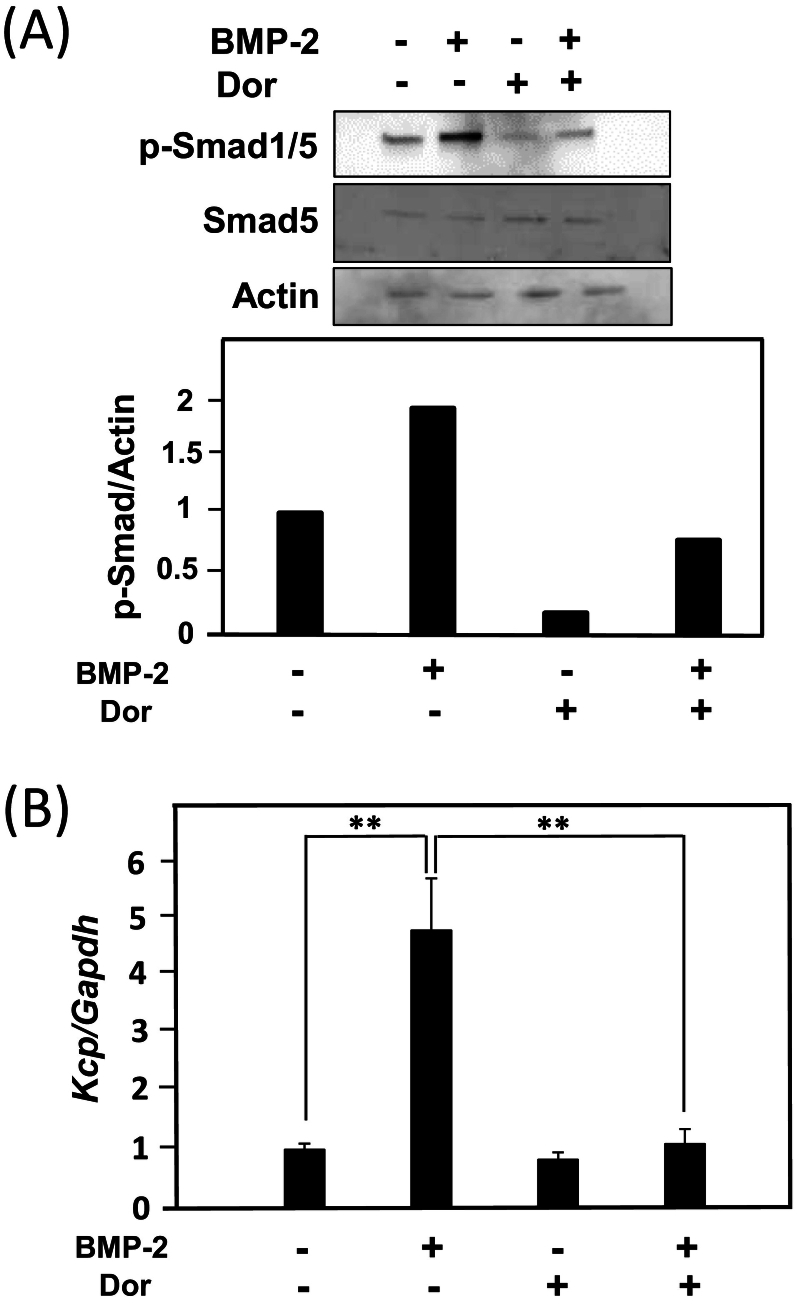
MC3T3-E1 cells were treated with 300 ng/ml of BMP-2 and/or 500 nM of Dorsomorphin (Dor) for 24 h. (A) Dorsomorphin blocked BMP-2-induced phosphorylation of Smad1/5. Using a control phosphorylated Smad 1/5/Actin value of 1, the expression level of phosphorylated Smad 1/5 was calculated as the relative ratio of Smad 1/5/Actin when treated with BMP-2 and Dorsomorphin. (B) The expression level of *Kcp* when treated with Dorsomorphin was examined. Using a control *Kcp/Gapdh* value of 1, the expression level of *Kcp* was calculated as the relative ratio of *Kcp/Gapdh* when treated with BMP-2 and Dorsomorphin. **p < 0.01 (Student's *t*-test).

Next, to investigate whether induction of *Kcp* expression by BMP-2 occurs via activation of SMAD signaling, the mode of *Kcp* expression following treatment with Dorsomorphin, an inhibitor of SMAD signaling (Yu et al., 2008), was examined. BMP-2 treatment resulted in phosphorylation of Smad1/5, with that activation inhibited by Dorsomorphin treatment (Fig. 3A). Furthermore, induction of *Kcp* expression by BMP-2 was suppressed by Dorsomorphin (Fig. 3B). BMP-2 is known to activate the canonical BMP-SMAD signaling pathway as well as the BMP-MAPK signaling pathway (Chen et al., 2012). To confirm whether MAPKs such as ERK1/2, JNK, and p38 MAPK are involved in BMP-2-induced *Kcp* expression, the level of *Kcp* expression with BMP-2 treatment in the presence of MAPK inhibitors was examined. Following pretreatment of MC3T3-E1 cells with 10 μM of U0126 (MEK inhibitor), SP600125 (JNK inhibitor), and SB203580 (p38 MAPK inhibitor), treatment with 300 ng/ml of BMP-2 was performed. Surprisingly, the MAPK inhibitors did not inhibit up-regulation of *Kcp* expression induced by BMP-2, but rather an increase in *Kcp* expression was observed (Suppl. Fig. 2). These results suggest that induction of *Kcp* expression is mediated through SMAD signaling and not MAPK signaling.

### 3.3 Knockdown of *Kcp* expression involved in promotion of osteoblast differentiation and mineralization by BMP-2

**Fig. 4:**
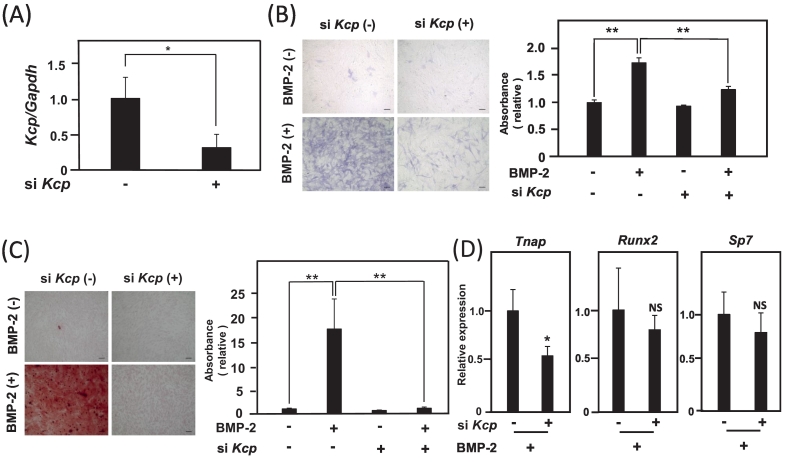
(A) The expression level of *Kcp* was examined when si *Kcp* was applied to MC3T3-E1 cells. *p < 0.05 (Student's *t*-test) (B) Measurements of ALP staining (*left panels*) and ALP activity (*right panels*). MC3T3-E1 cells were treated with 300 ng/ml of BMP-2 and si *Kcp* for three days, then the effects on osteoblast differentiation were determined. Scale bar shown represents 100 μm. **p < 0.01 (Student's *t*-test) (C) MC3T3-E1 cells were stained with Alizarin red in the presence or absence of 300 ng/ml of BMP-2 and si *Kcp* for seven days. Alizarin red dye associated with the cells was dissolved and quantified by measurement of absorbance at 570 nm. Scale bar shown represents 100 μm. **p < 0.01 (Student's *t*-test) (D) MC3T3-E1 cells were treated with 300 ng/ml of BMP-2 for two days and si *Kcp*, then expressions levels of osteoblast differentiation marker genes (*Tnap*, *Runx2*, *Sp7*) were examined using quantitative RT-PCR. *p < 0.05, NS: not significant.

Finally, to investigate how induction of *Kcp* expression by BMP-2 in osteoblasts affects osteoblast differentiation induced by BMP-2, the effects of BMP-2 on osteoblast differentiation when *Kcp* expression was suppressed were examined. si *Kcp* treatment suppressed *Kcp* expression by approximately 70 % (Fig. 4A). Analyses of ALP staining to determine activity showed that BMP-2-induced osteoblast differentiation was reduced by suppression of *Kcp* expression (Fig. 4B). In addition, mineralization in osteoblasts treated with BMP-2 was suppressed by si *Kcp* (Fig. 4C), while expression of Alp (*Tnap*) was also suppressed by si *Kcp*, whereas expressions of transcription factors for osteoblast differentiation were not affected (Fig. 4D). Based on these results, it is considered possible that induction of *Kcp* expression is involved, at least in part, in promotion of osteoblast differentiation and mineralization by BMP-2.

## 4 Discussion

The present results indicate that Kcp provides positive feedback for promotion of osteoblastic differentiation by BMP-2 in osteoblasts. BMP antagonists are induced by BMPs such as *Noggin*, which is known to block BMP signaling induced by BMP-2 (Gazzerro et al., 1998). Furthermore, BMP-2 has been found to up-regulate the expression of *Gremlin 2*, a negative regulator of BMP function during osteoblast differentiation (Suzuki et al., 2012). That study also noted regulation of BMP activity by Noggin and Gremlin 2 following a negative feedback system, which may have important roles for protecting bone metabolism from excessive activation by BMP. On the other hand, *Nephronectin*, thought to promote osteoblast differentiation, has been shown to be induced by BMP-2 in C2C12 cells (Kurosawa et al., 2016). Thus, BMP signaling is considered to be controlled by both positive and negative feedback regulation during the process of osteoblast differentiation.

In the present study, *Kcp* expression induced by BMP-2 was shown to be suppressed by the SMAD signaling inhibitor Dorsomorphin, thus it is speculated that *Kcp* induction by BMP-2 is mediated through SMAD signaling. BMP signaling has been shown to activate MAPK in osteoblasts in addition to SMAD-dependent signaling (Weiskirchen et al., 2009), such as previous findings indicating that Osterix, a key transcription factor for osteoblast differentiation, is induced by BMP-2 through p38-mediated MAPK signaling (Celil et al., 2005). However, the present results showed that MAPK activation did not inhibit *Kcp* expression induced by BMP-2, rather an increase in *Kcp* expression was observed. A future study that examines regulation of *Kcp* expression through MAPK signaling is needed.

Physiological functions of Kcp have been shown to inhibit renal fibrotic disease (Lin et al., 2005b), decrease acute and chronic renal injury (Soofi et al., 2013), and suppress aging- and high-fat diet-induced nonalcoholic fatty liver disease (Soofi et al., 2017). Recently, Ye et al. reported that Kcp knockout mice demonstrated cardiac aging (Ye et al., 2023). In the present study, bone morphometric analysis showed no obvious differences in trabecular bone obtained from wild-type and Kcp knockout mice (Suppl. Fig. 3). Additional physicological study findings are needed to clarify whether up-regulation of *Kcp* expression by BMP-2 is involved in bone metabolism.

## 5 Conclusion

In the present study, induction of *Kcp* by BMP-2 was found to provide positive feedback for promotion of osteoblastic differentiation in osteoblasts. Additional research aimed at hard tissue regeneration as well as development of methods for analysis, such as fracture and bone defect repair models, will be conducted in future studies in order to elucidate the physiological mechanism of BMP signal activation by Kcp.

## Financial support statement

This work was supported in part by a grant from 10.13039/501100001691JSPS Kakenhi (21K09833).

## CRediT authorship contribution statement

**Kazuki Toba:** Writing – original draft, Formal analysis, Data curation, Conceptualization. **Atsushi Yamada:** Writing – review & editing, Writing – original draft, Funding acquisition, Formal analysis, Data curation, Conceptualization. **Kiyohito Sasa:** Formal analysis, Data curation. **Tatsuo Shirota:** Writing – review & editing, Supervision. **Ryutaro Kamijo:** Supervision, Conceptualization.

## Declaration of competing interest

None of the authors have conflicts of interest to declare regarding the contents of this article.

## Data Availability

The data that has been used is confidential.

## Appendix A Supplementary data

Suppl. Fig. 1 BMP-2 (300 ng/ml) was added to mouse skull-derived primary cultured osteoblasts for 24 h, then the expression level of *Kcp* was examined. Using a control *Kcp/Gapdh* value of 1, the relative ratio of *Kcp/Gapdh* when treated with BMP-2 was determined. *p < 0.05 (Student's *t*-test) Suppl. Fig. 2 *Kcp* expression level following treatement with MAPK signaling inhibitors U0126, SP600125 (SP), and SB203580 (SB). Using a control *Kcp/Gapdh* value of 1, the expression level of *Kcp* was calculated as the relative ratio of *Kcp/Gapdh* when treated with BMP-2 (300 ng/ml) and the inhibitors (10 μM). *p < 0.05, **p < 0.01 (Student's *t*-test) Suppl. Fig. 3 (A) sections of Femur sections of wild-type (WT) and Kcp knockout (KO) mice obtained after seven weeks, and subjected to Villanueva bone staining. Scale bars: 500 μm. (B) Parameters examined related to bone structure included total bone volume per tissue volume (BV/TV, %), trabecular thickness (Tb.Th, μm), osteoid thickness (O.Th, μm), number of osteoblasts per bone surface (N.Ob/BS, N/mm), and number of osteoclasts per bone surface (N.Ob/BS, N/mm). NS: not significant, Mu: multinuclear cells, Mo: mononuclear cells Image 1
