# Supplementary figures and images for: Expression of Kielin/chordin-like protein is regulated by BMP-2 in osteoblasts
Source: Bone Rep. 2024 Jul 20;22:101793. doi: 10.1016/j.bonr.2024.101793 (PMC11321374; doi:10.1016/j.bonr.2024.101793)

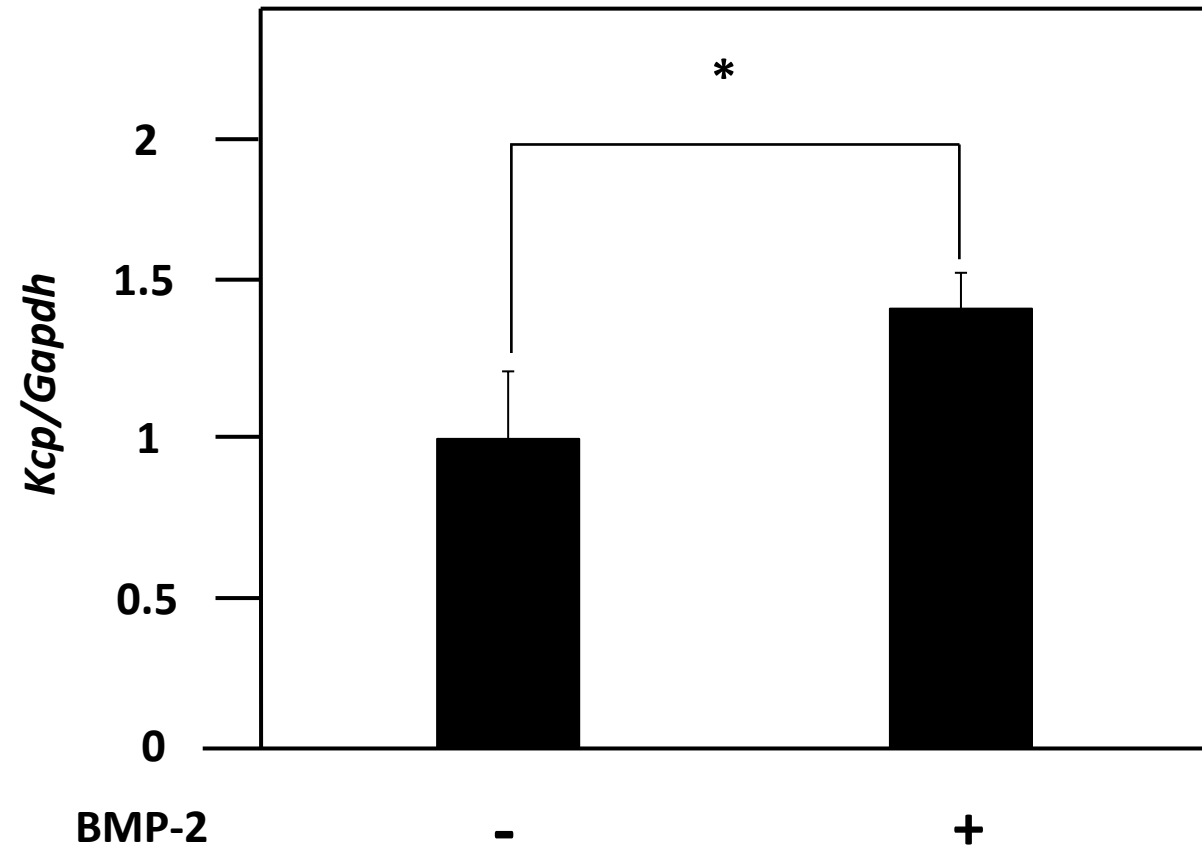

Suppl. Fig. 1. Toba K. *et al.*

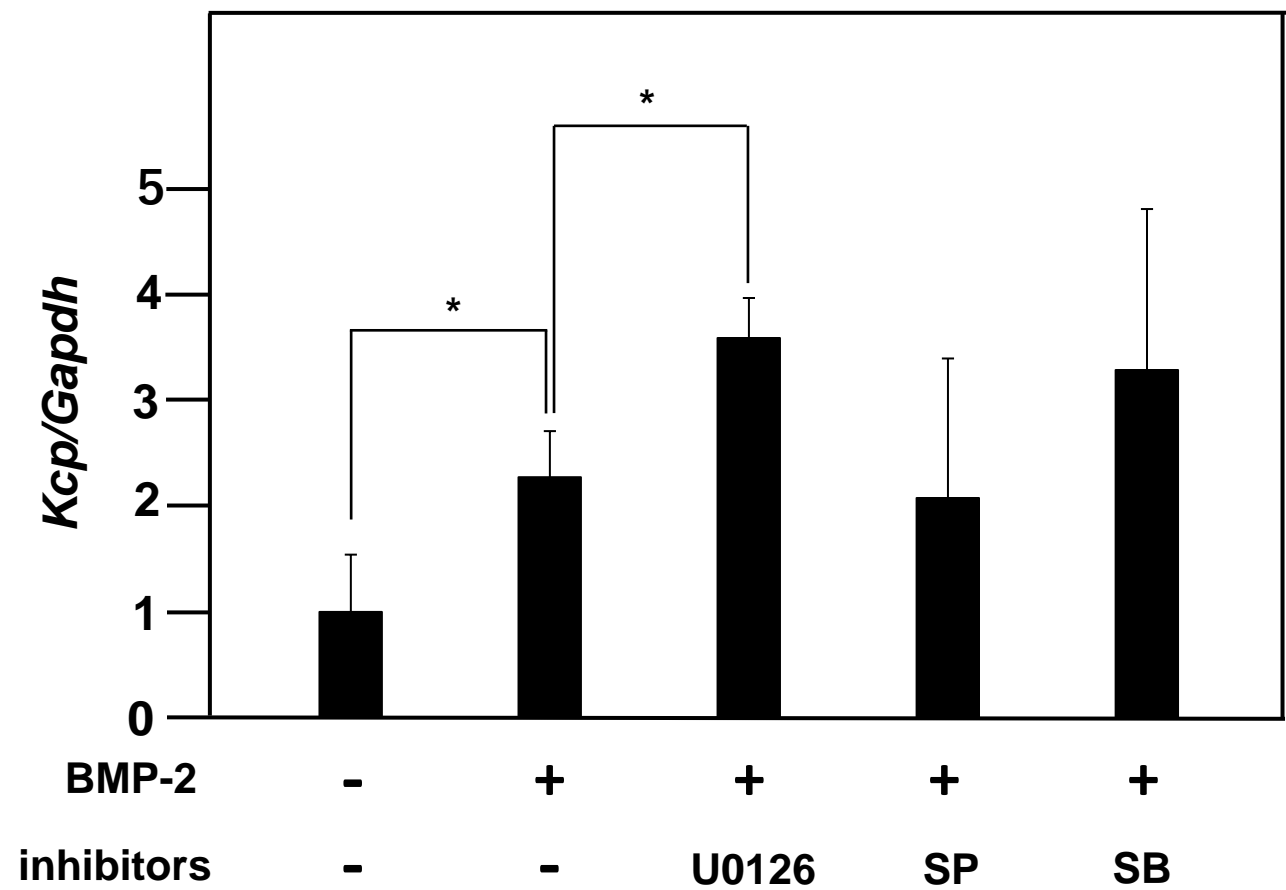

Suppl. Fig. 2. Toba K. *et al.*

(A)

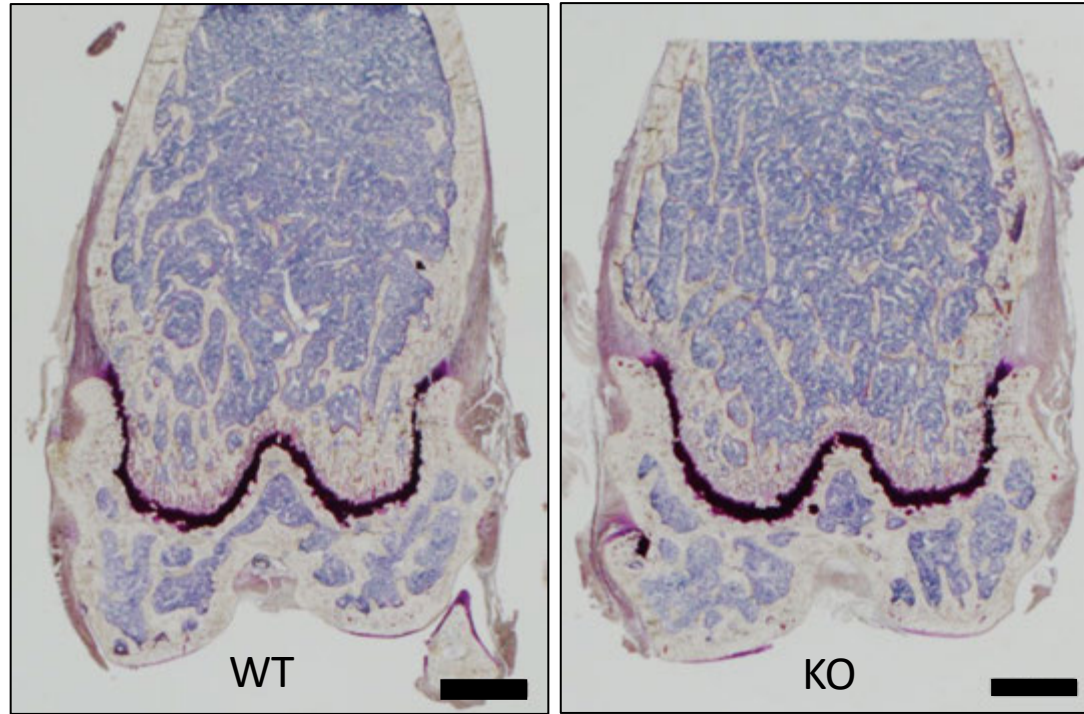

(B)

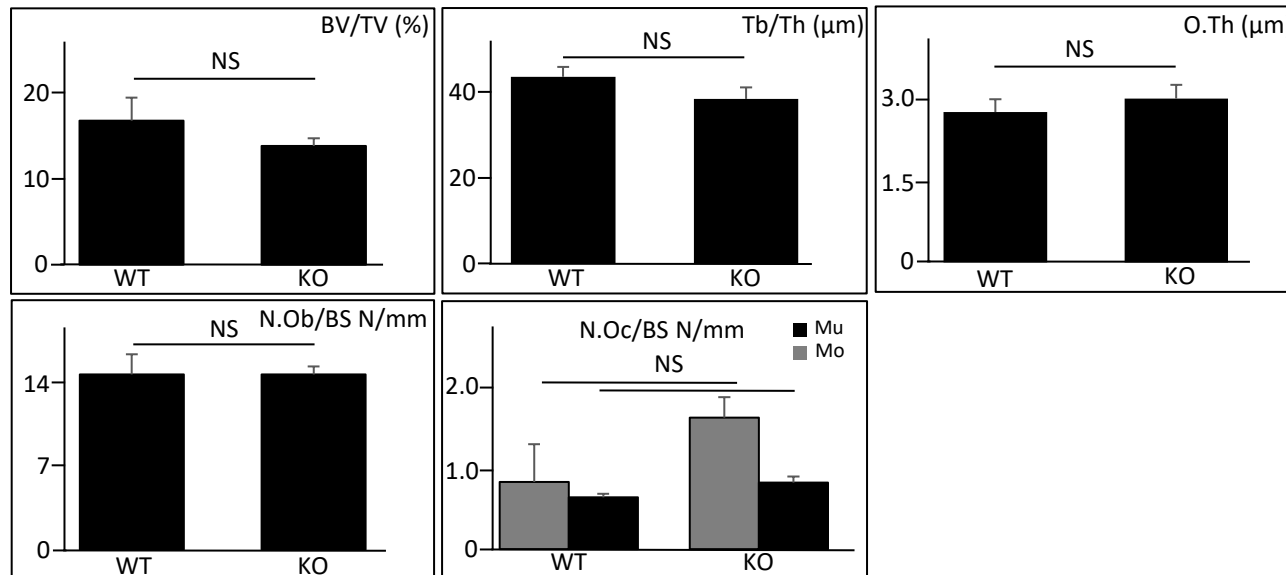

Supplement: Supplementary file 1 — Suppl. Fig. 1 BMP-2 (300 ng/ml) was added to mouse skull-derived primary cultured osteoblasts for 24 h, then the expression level of Kcp was examined. Using a control Kcp/Gapdh value of 1, the relative ratio of Kcp/Gapdh when treated with BMP-2 was determined. *p < 0.05 (Student's t-test) Suppl. Fig. 2 Kcp expression level following treatement with MAPK signaling inhibitors U0126, SP600125 (SP), and SB203580 (SB). Using a control Kcp/Gapdh value of 1, the expression level of Kcp was calculated as the relative ratio of Kcp/Gapdh when treated with BMP-2 (300 ng/ml) and the inhibitors (10 μM). *p < 0.05, **p < 0.01 (Student's t-test) Suppl. Fig. 3 (A) sections of Femur sections of wild-type (WT) and Kcp knockout (KO) mice obtained after seven weeks, and subjected to Villanueva bone staining. Scale bars: 500 μm. (B) Parameters examined related to bone structure included total bone volume per tissue volume (BV/TV, %), trabecular thickness (Tb.Th, μm), osteoid thickness (O.Th, μm), number of osteoblasts per bone surface (N.Ob/BS, N/mm), and number of osteoclasts per bone surface (N.Ob/BS, N/mm). NS: not significant, Mu: multinuclear cells, Mo: mononuclear cells [file mmc1.pdf]
